# The Choice of Names

**Published:** 1919-04-05

**Authors:** 


### THE CHOICE OF NAMES.

The selection and application of names is a more delicate art than is commonly imagined, and the consequences of the choice may be far reaching. Used at first as a mere convenience, or as a symbol, a name often in time comes to exercise control over the ideas of many who use it, and thus it saves "talkative people the trouble of thinking." Not a few of the confusions both of general and technical discussions arise from misunderstandings created by badly selected names. One of the main faults in the selection of names is the introduction of terms which involve hypotheses or speculations, and in this way erroneous suggestions and conclusions have in individual instances been carried over the life history of generations. The strict rule ought to be that any new name proposed for introduction into scientific nomenclature must either be a purely arbitrary choice or must merely intimate the fact to which it is attached. In any circumstances it must not involve a speculative or theoretical interpretation of the origin or nature of this fact. Thus such a term as the "X disease" is good, as is also measles or smallpox. Each such name is a purely arbitrary choice, and each is fixed to a certain body or series of facts. The facts will remain whatever changes of opinion relative to them may occur, and as the name does not enclose any suggestion of opinion, it can never be a source of confusion or error. From the same point of view may be defended the practice of attaching to any newly discovered disease the name of the discoverer. Addison's anæmia, Bright's disease, and so on may claim to be names free from the confusing influence of opinion, and to this extent they are good. The late Sir Jonathan Hutchinson used to claim that not the physician who described the new symptom or disease, but the patient who produced it, should have the honour of fixing his name to the peculiarity, and thus he recorded notes under such headings as "Mrs. H.'s legs" and "Mr. A.'s skin eruption." Such a practice is obviously based on the policy here advocated —namely, that scientific names shall not go beyond the record of the facts.

As a contrast to the above, take the nomenclature which is commonly cultivated in the description of chronic diseases of the joints. Such names as "rheumatic," "rheumatoid," "gouty," and others are freely employed, and each, when selected, includes a doctrine of causation which is nowadays regarded as of very doubtful accuracy. Books and articles and treatises without number have been written around these terms and about their application in the field of diagnosis. Yet in reference to the causation of the conditions to which they are mostly applied, we are so far almost entirely in the dark. The position used to be put in a somewhat cynical fashion by a well-known teacher of medicine, who, when discussing certain chronic diseases of the joints, used to say, "Some authorities, gentlemen, say that these disturbances are rheumatism; others say they are gout, while prudent persons who know how to make the best of both worlds call them rheumatic gout." On the other hand, a physician whose authority was, and indeed still is, widely recognised, found one of his favourite doctrines in the proposition that there is no such disease as rheumatic gout, meaning, presumably, that rheumatism and gout never exist at one and the same time in one and the same patient. A discussion of the same order spreads over many phenomena other than those occurring in connection with the joints, and battles are conducted on behalf of names which suggest a knowledge of causes far in advance of the facts. Such are some of the ills consequent on the selection of terms which involve speculative considerations, and which consequently lead to confusion and debates that might well have been avoided.

Another illustration of the same order, though perhaps in a less serious field, is found in connection with the term "chemist," as this is used at the present day. On the one hand, this is the term applied to highly skilled laboratory workers or investigators in the department of chemistry. On the other hand, it commonly signifies a person who keeps open shop for the dispensing of physicians' prescriptions and for the sale of medicinal preparations. Some of these preparations consist of dried vegetable products, or *drugs* in the strict meaning of the term, and hence the person dealing in them is reasonably called a *druggist*, while others, of mineral origin mainly, received, and popularly indeed still receive, the name of *chemicals*, so that the individual who dealt with these became spoken of as a *chemist*. Time and the increase of knowledge have upset this sharp opposition, and have given us many "chemicals" of vegetable origin. But the names which grew out of this opposition still remain, and may be seen in the trade announcement, "Pure drugs and chemicals," and in the title "chemist and druggist." The scientific worker in chemistry appeared later in the field, and when he arrived he found that his natural designation of "chemist" had already been applied to another worker, and under this bond he remains to this day. Naturally he would fain take the title appropriate to his calling, and would persuade the "chemist and druggist" to become "pharmacist"; and we feel to his wishes every goodwill. But to change a name established by long habit is almost impossible, and the experience here quoted may well enforce the lesson that the choice of a name is a serious undertaking.

